# Retrograde Ureteric Stents via an Ileal Conduit

**DOI:** 10.1155/2011/904017

**Published:** 2011-07-07

**Authors:** Andrew Jack, Brent E. Burbridge

**Affiliations:** ^1^College of Medicine, University of Saskatchewan, Saskatoon, SK, Canada S7N 0W8; ^2^Medical Imaging Department, College of Medicine, University of Saskatchewan, Saskatoon, SK, Canada S7N 0W8; ^3^Medical Imaging Department, Royal University Hospital, 103 Hospital Drive, Saskatoon, SK, Canada S7N 0W8

## Abstract

Patients having undergone pelvic exenteration with urinary diversion can present with short- and long-term complications such as ureteral strictures, anastomotic leakage, calculi, or fluid collections (abscess, urinoma, lymphocele, or hematoma). A dehiscence resulting in a perineal urinary fistula is an uncommon late complication of urinary diversion surgery; surgical treatment for this complication is less desirable because of postsurgical or radiation-induced pelvic changes that can occur. As a result, nephrostomy or antegrade stenting of the kidneys is more viable. Retrograde ureteric stent insertion is discussed in relation to a patient suffering from ileal conduit dehiscence. The presence of these stents probably helped diminish the potential for complications during subsequent nephrostomy tube insertion.

## 1. Introduction

Surgeons are usually very reluctant to operate on delayed complications of ileal conduits due to the postoperative and postradiation changes impeding the potential for successful repair or reconstruction. Some of these complications can be managed by interventional radiology in a minimally invasive manner [1].

Urinary diversion (UD) following radical cystectomy is a common surgical procedure for malignancies involving the urinary bladder. UD can be classified into two large groups based upon urinary continence or incontinence [2]. The incontinent UD options, such as the ileal conduit, remain the gold standard [3]. 

Although the ileal conduit is heavily relied upon for UD, significant mortality and morbidity (51.2%) still exist with its formation [4]. Among the most common early-onset complications for UD are anastomotic leakage, abscess formation, metabolic acidosis, fistulization, and infection. Long-term UD complications, however, often revolve around the ureteric anastomoses and conduit integrity [4].

In the event that long-term UD complications do occur, surgical intervention is generally avoided due to postoperative pelvic changes and/or previous pelvic radiation treatment [4]. Interventional radiology may be asked to be involved in the management of these patients. Minimally invasive interventional treatment options may include percutaneous nephrostomy, ureteric dilation, and placement of ureteral or ureteroileal stents. This case demonstrates that retrograde ureteric stent placement is possible in nonhydronephrotic kidneys for a patient with an ileal conduit.

## 2. Case Report

This 68-year-old male underwent pelvic exenteration, right lower quadrant ileal conduit creation, and left lower quadrant colostomy for the treatment of rectal carcinoma. Four years after these surgical procedures, the patient presented with vague lower abdominal and perineal pain and decreased ileal conduit output for four days. In addition, he related that he was experiencing persistent drainage of cloudy, yellow fluid from the perineum for four weeks.

On physical examination, the patient was found to have a wet perineum with a visible draining sinus in the ventral aspect of the anal scar.

**Figure 1:**
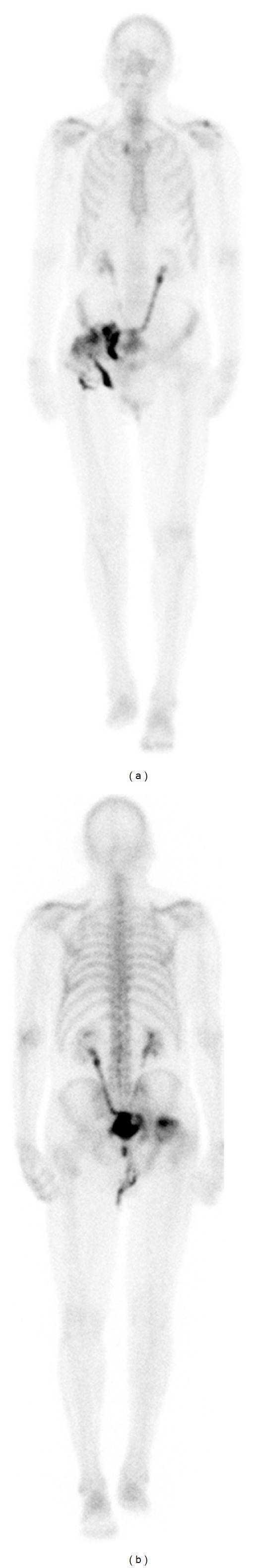
Nuclear medicine bone scan demonstrating an ileal conduit leak into the deep pelvis. No metastatic disease detected (anterior view on the left and posterior view on the right).

**Figure 2:**
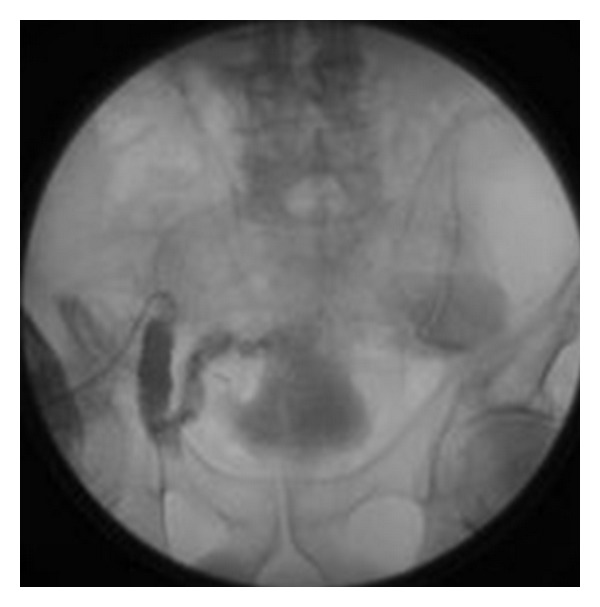
Loopogram demonstrating proximal contrast leakage from the conduit without visualization of either ureter.

Renal ultrasound was performed and revealed normal kidneys with no evidence of hydronephrosis. A nuclear medicine bone scan was negative for metastatic disease, but it was evident that the ileal conduit was leaking urine into the deep pelvis (Figure 1). A retrograde ileal conduit examination, with water soluble contrast, revealed contrast flowed promptly from an open end of the conduit intoa pelvic fluid collection. Neither ureter could be visualized as the contrast preferentially flowed into the pelvis (Figure 2). The findings were considered to be consistent with a proximal ileal conduit dehiscence and extravasation of contrast into a pelvic cavity.

Urology consulted interventional radiology to discuss placement of bilateral nephrostomy catheters, followed by antegrade ureteric stents, in an attempt to divert urine away from the conduit dehiscence. 

In the absence of hydronephrosis, nephrostomy insertion was deemed to be too technically difficult and would require multiple needle punctures of the kidneys while attempting to gain access to the collecting system. In addition, the collapsed collecting systems would present a very confined space in which to attempt to maneuver needles, guide wires, and catheters. Because of the probability of procedure technical failure, and heightened concerns about procedural complications, another treatment option was felt to be necessary. It was decided that attempting retrograde ureteric stent placement in the interventional radiology suite was the most appropriate solution.

**Figure 3:**
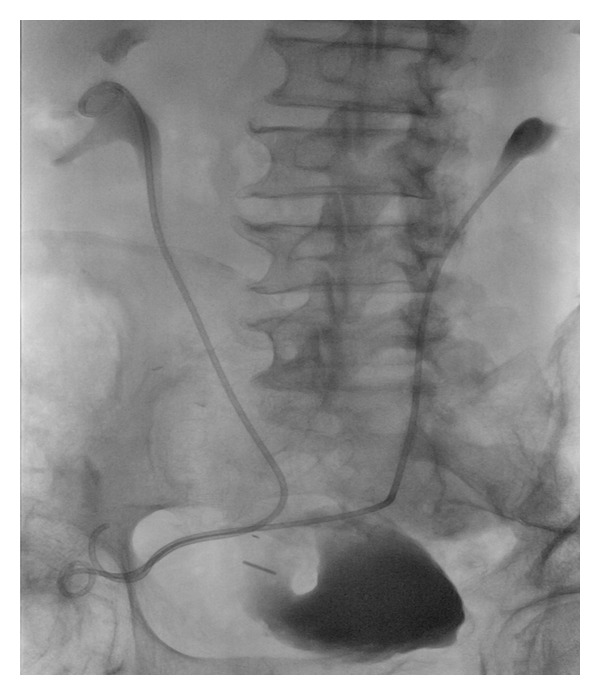
Contrast study after stent placement demonstrating right and left ureteric stents extending from renal pelves, through each ureter and exiting through the conduit ostomy.

The conduit ostomy bag was removed and the ileal conduit was catheterized retrograde with a 5F Kumpe angiographic catheter (Cook Canada, Inc.). Under fluoroscopic guidance the Kumpe catheter was used to probe for the ureteric anastomoses within the ileal conduit. The left ureter was initially catheterized with the Kumpe catheter. With the assistance of a “0.035”, 15 mm, guide wire (Cook Canada, Inc.) it was possible to implant an Inlay Optima 8F, double J, ureteric stent (Bard, Inc.) in a retrograde fashion. The proximal J of the stent was deployed in the renal pelvis, and the distal J was deployed just external to the skin surface of the ileal conduit. An 8F, double J, InLay ureteric stent was also implanted on the right side, in the same manner. The patient tolerated the procedure without difficulty or complication. The results of the stent procedure are shown in Figure 3. 

The patient was discharged the next day for outpatient management. It was hoped that the stents would divert urine away from the conduit reservoir and allow the conduit dehiscence to heal. However, despite this conservative therapy and the presence of the ureteric stents the patient required nephrostomy tubes. The ureteric stents were utilized for this procedure as they were injected retrograde to opacify and dilate the renal collecting systems bilaterally for nephrostomy insertion.

## 3. Discussion

The placement of nephrostomy tubes in this individual was initially avoided as it would have been technically very difficult, and potentially associated with an increased complication profile because of the complexities associated with nephrostomy insertion in a nonhydronephrotic kidney.

The retrograde ureteric stent insertion was most certainly greatly facilitated by the patent ureteric anastomotic orifices and the lack of hydronephrosis. If the patient had hydronephrosis with distal ureteric anastomotic strictures, it probably would have been very difficult to catheterize the renal pelves retrograde via the ileal conduit.

Applbaum et al. demonstrated successful retrograde ureteric stent placement in the intact ileal conduit in 14/17 (82.3) ureters. They concluded that “in patients with ileal conduits in whom access to the urinary tract is necessary, retrograde catheterization is a safe and painless alternative that should be attempted before percutaneous nephrostomy” [5].

Zaleski et al. did not demonstrate similar success as they were only able to perform retrograde ureteric stent placement in only 6/13 attempts (46%) [6].

Neither of these case series evaluated retrograde stent placement in the setting of conduit dehiscence, and opacification of the collecting system for the purpose of delayed nephrostomy was not employed.

Our experience demonstrates that if ureteric stents are required for a patient with an ileal conduit, in the absence of hydronephrosis, the retrograde placement of these stents is technically feasible. In addition, if nephrostomy tube placement is necessary in the future, the distal ends of the ureteric stents can be injected with contrast to opacify the renal collecting system, facilitating nephrostomy deployment.
